# A 3-year prospective randomized clinical trial of alveolar bone crest response and clinical parameters through 1, 2, and 3 years of clinical function of implants placed 4 months after alveolar ridge preservation using two different allogeneic bone-grafting materials

**DOI:** 10.1186/s40729-022-00402-w

**Published:** 2022-02-01

**Authors:** Önder Solakoğlu, Duygu Ofluoğlu, Heidi Schwarzenbach, Guido Heydecke, Daniel Reißmann, Sertan Ergun, Werner Götz

**Affiliations:** 1 Center for Dental and Oral Medicine, University Medical Center Hamburg-Eppendorf, Martinistr. 52, 20246 Hamburg, Germany; 2 Department of Oral Medicine and Maxillofacial Surgery, Faculty of Dentistry, Istanbul University, Istanbul, Turkey; 3 Institute of Tumor Biology, University Hospital Hamburg-Eppendorf, Hamburg, Germany; 4 Laboratory for Oral Biologic Basic Science, Department of Orthodontics, University of Bonn, Bonn, Germany; 5 Department of Prosthodontics Dental, University Medical Center Hamburg-Eppendorf, Hamburg, Germany; 6 Specialty Dental Practice Limited to Periodontology and Implant Dentistry, Hamburg, Germany

**Keywords:** Allogeneic bone graft, PRGF, Pericardium membrane, Implants, Extraction socket, Alveolar ridge preservation

## Abstract

**Purpose:**

The aim of this study was to longitudinally evaluate changes in alveolar bone crest (ABC) levels and differences in resorption rates (RR) between the tested grafting materials following alveolar ridge preservation (ARP) after tooth extraction after 1, 2, and 3 years (T1–T8) of clinical function.

**Methods:**

Patients were randomly assigned to two different bone allografts (group 1 maxgraft^®^, group 2 Puros^®^) for ARP. Non-restorable teeth were minimal traumatically extracted. Sockets were augmented with the tested materials and covered with a pericardium membrane. After 4 months of healing, 36 implants were placed and sites were clinically and radiographically monitored in the mesial (ABC-M), the distal (ABC-D, T1–T8), the bucco-lingual (ABC-BL), buccal (ABC-B) and oral (ABC-O) aspect (T1–T4).

**Results:**

Changes in (ABC-M), (ABC-D), (ABC-BL), (ABC-B), and (ABC-O) levels showed statistically highly significant differences between T1 and T2 for both bone allografts (*p* < 0.001). Changes at the ABC-M and ABC-BL levels between T2 and T3 of group 1 showed a statistically significant difference (*p* < 0.001). Both groups achieved and maintained increased ABC levels without statistically significant differences throughout the monitoring periods of 1–3 years (T6–T8) of clinical function. No failures or adverse events were observed.

**Conclusions:**

To the best of our knowledge, this study is within its limitations the first study to directly compare ABC-changes and differences in RR of two different allogeneic grafting materials for a period of 3 years after ARP. It was demonstrated to be, despite significant differences in RR, a successful method of preserving increased ABC levels through 1, 2, and 3 years of clinical function.

*Trial registration* DRKS00013010, registered 07/30/2018, http://apps.who.int/trialsearch

## Background

Alveolar bone is maintained by the presence of teeth in the jaw. Stimulation to the supporting jawbone provided by tooth roots during function helps preserving alveolar ridge dimensions in accordance with Wolff’s law [1, 2]. Unfortunately, teeth may be lost due to trauma or local and systemic diseases, or they may be even congenitally absent. It is well known that the bone resorption of the alveolar part of the jaws after tooth extraction is accelerated in the first 6 months after extraction, which is followed by gradual remodeling that may include major changes in size and shape [3, 4]. These very time-dependent changes mainly concern height and width reduction of the alveolar bone. Up to 2–4 mm horizontal loss [3, 5, 6], (29–63%) and 1 mm (11–22%) vertical loss of the alveolar ridge could be seen 6 months after tooth extraction, whereas nearly 6 mm of buccal alveolar bone loss can be expected by the end of the first year after extraction [3]. A previous meta-analysis concluded that an average reduction of 3.87 mm in the bucco-lingual ridge thickness and a vertical mid-buccal resorption of 1.67 mm can be expected following unassisted socket healing [7]. Numerous factors can exacerbate the resorption process, such as the cause of tooth failure for example following an inflammatory process like periodontal disease or an endodontic complication, the degree of extraction trauma, the location of the defect site, bone density, the presence of metabolic diseases, and the type of prosthesis used to restore the missing dentition [8, 10–14]. These three-dimensional changes can lead to difficulties in implant positioning, resulting in esthetic compromises or even an impossibility of implant placement.

In order to prevent bone resorption and to prevent post-extraction bone loss as well as to promote bone regeneration of the residual alveolar socket, the use of grafting materials for post-extraction socket preservation has been described under different designations such as ‘socket preservation’, ‘ridge preservation’, and/or ‘alveolar ridge preservation’ (ARP) [11, 12, 15–19]. Various bone grafts and substitutes such as autogenous bone, allografts, xenografts, and alloplast materials in combination with or without barrier membranes have been investigated and are well documented in the literature in various animal and human studies for the augmentation of extraction sockets [20]. Among these grafting materials, autogenous bone is accepted as the ‘gold standard’ because of its osteogenic, osteoinductive, and osteoconductive properties [21, 22]. Unfortunately, its practical use is limited due to factors like the need of a second surgery in the donor site, an increased risk of postoperative infection, and increased patient morbidity. Therefore, allogeneic bone-grafting materials became widely used as a bone substitute.

Allografts are tissues taken from individuals of the same species. They provide type 1 collagen and bone morphogenic proteins (BMPs), which could be osteoinductive compounds and encourage new bone formation 23. The advantage of allografts is that they are readily available without a second surgical site and provide osteoinductive as well as osteoconductive properties and become resorbed within a reasonable amount of time without causing inflammatory reactions and promote new bone formation [24–26].

In a previous histological and immunohistochemical study, we could demonstrate that the commercially available allogeneic bone-grafting materials used in the present study showed equivalent results of clinical outcome, bone formation and lack of immunological potential in alveolar ridge augmentation procedures [26]. Similar results were reported by other scientific groups [27–29].

Furthermore, covering the graft material with a membrane by the time of ARP influences the amount of newly formed bone, leading to a greater amount of vital bone [30]. As barrier membrane we used the Jason^®^ membrane (Botiss biomaterials GmbH, Germany part of Straumann group, Basel, Switzerland), which is a native porcine collagen membrane originating from pericardium, which provides a strong multidirectional-linking of the collagen network and a long-lasting barrier function for 12–24 weeks.

It has been shown that the ridge preserved areas demonstrated greater bone height, bone width, and total bone volume when compared with the areas of unassisted naturally healed bone at the time of implant placement using 2- and 3-dimensional radiographs [31].

**Fig. 1:**
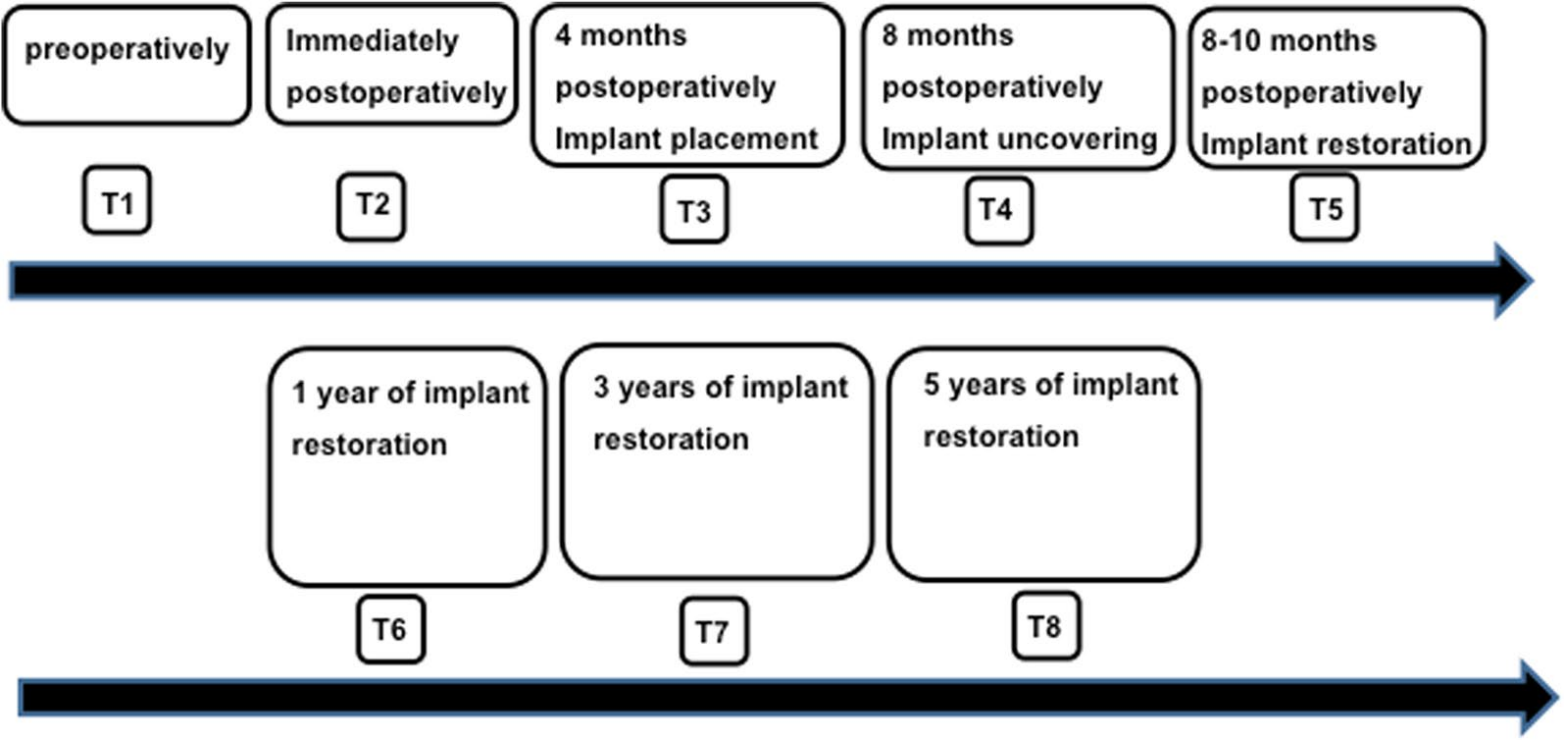
Flowchart of the study design showing the eight different time points of measurements of alveolar bone crest levels

In the present RCT, we longitudinally evaluated over a period of 3 years of clinical functioning alveolar bone crest (ABC) levels around dental implants and adjacent teeth using radiographic reference points at the mesial (ABC-M) and at the distal (ABC-D) aspects at eight different time points (T1–T8). Furthermore, we clinically evaluated the bucco-lingual changes of the dimensions of alveolar bone crest width (ABC-BL) and the buccal and oral height of the alveolar crest (ABC-B and ABC-O) at 4 different timepoints (T1–T4) in order to account for changes due to the early resorption of the buccal and/ or the oral plate of bone following tooth extraction [32]. We also carried out a clinical 6-point charting at 4 different time points following implant restoration (T5–T8) (Fig. 1).

The hypothesis was tested, if there are any significant differences in the alveolar bone crest dimensions at the mesial and the distal radiographic measurement points as well as the clinically measured alveolar bone crest width (ABC-BL) and alveolar bone crest height (ABC-B and ABC-O) between the two tested materials, a solvent-dehydrated allogeneic material from a single donor (Puros^®^) and a freeze-dried bone allograft material pooled from multiple donors (maxgraft^®^) in combination with the use of a resorbable pericardium barrier membrane (Jason^®^ membrane).

## Methods

### Study design and population

As a prospective randomized parallel-group clinical trial, this publication was written on the basis of CONSORT guidelines [33]. Thirty-six patients, who presented at one private practice limited to periodontics and implant dentistry (FPI-Hamburg, Dr. Önder Solakoglu) with the need for a single tooth extraction because of hopeless prognosis of the individual tooth and subsequent implant placement, were enrolled in this randomized prospective clinical trial between July 2016 and February 2017. General exclusion criteria for enrollment in this study were skeletally immature patients, persons with uncontrolled systemic diseases, a history of radio- and/or chemotherapy, pregnancy, active periodontal disease, poor oral hygiene and smoking habit. Site-specific exclusion criteria were fully intact extraction sockets (4-wall defects) as well as 1-wall defects, at least one wall of the extraction socket had to be compromised (3-wall or 2-wall defects). The general health history and oral hygiene status of each patient was carefully reviewed, and the mobility status, presence of furcation defects, periodontal defects, occlusal contact, endodontic status of the non-restorable tooth as well as the type of crown retention of the later implant restoration (screw-retained or cement-retained) were evaluated and noted. Oral hygiene procedures were reviewed with the patient, then restorative options, study goals, and requirements for study participation were thoroughly discussed, and all questions were addressed. Prior to study inclusion, each patient provided signed informed consent. After inclusion, patients were clinically and radiographically evaluated with standardized periapical radiographs using a Rinn extension cone parelleling X-ray holder with an occlusal bite index (Rinn XCP, Dentsply Rinn, York, PA, USA). Clinical photographs were also taken. Each subject was monitored by the same blinded investigator throughout the study period. Patients were clinically and radiographically reevaluated after 1 year (T6), 2 years (T7), and 3 years (T8) following restoration and clinical functioning of the implant restoration. All patients gave their informed consent and all patients completed the study successfully and were available for follow-up visits. No adverse events were recorded.

All procedures performed in this study were in accordance with the ethical standards of the institutional and/or national research committee and with the 1964 Helsinki declaration and its later amendments or comparable ethical standards and were approved by an ethics committee (Hamburg Medical Association, Germany, no. PV5211) and the study was registered with the German Register for Clinical Trials (DRKS no.: 00013010). Consent was obtained from all patients for publication of this study and any accompanying images and data.

As allografts for augmentation of the extraction sockets, we used maxgraft^®^ cancellous granules in group 1 and Puros^®^ cancellous granules in group 2, each in half of all patients (Puros^®^ Cancellous Particulate Allograft, Zimmer Biomet Dental, Palm Beach Gardens, FL, USA; maxgraft^®^ cancellous granules, botiss biomaterials GmbH, Berlin, Germany, part of Straumann Group, Basel, Switzerland). Following inclusion in the study and equal distribution of the patients regarding age, gender, location of the tooth to be extracted, and implant brand, the participants were randomly assigned to one of the two groups by a blinded clinician not involved in this study and not involved in the specialty dental office by drawing a sealed envelope indicating inclusion in group 1 or group 2.

### Surgical procedures

Following tooth extraction, the defects of the different socket walls [mesial (ABC-M), distal (ABC-D), buccal (ABC-B), oral (ABC-O)] as well as the bucco-lingual width (ABC-BL) of the extraction sockets were clinically measured (T1). These measurements were repeated immediately following bone augmentation (ARP, T2), at implant placement 4 months later (T3), and another 4 months later at implant uncovery (T4) using a sterilized calibrated implant probe (Hu-Friedy Mfg. Co., LLC. European Headquarters Astropark Lyoner Str. 9. D-60528 Frankfurt am Main Germany, Global Headquarters, Chicago, Illinois, USA) as well as sterilized precision dental caliper (Dental caliper 800/5, Otto Leibinger GmbH, Griesweg 27, 78570 Mühlheim, Germany) and were statistically evaluated in order to detect statistically significant changes of alveolar bone crest levels.

#### Surgical phase 1: tooth extraction and ridge preservation (T1 and T2)

Immediately before tooth extraction, 60–100 mL of venous blood was drawn from the patient’s arm and processed in order to extract plasma rich in growth factors (PRGF) according to previously described instructions from the manufacturer of the processing unit [34–36] (Endoret PRGF Technology, BTI Biotechnology Institute, S.L., Miñano, Spain). Briefly, the collected venous blood was citrated and centrifuged at 580 *g* for 8 min, then plasma fractioning was performed to separate the first top fraction of plasma (fraction 1; F1) from the 2 mL of plasma (fraction 2; F2), the richest in platelets, and located just above the buffy coat [37]. Platelet activation was achieved by adding 50 ml of 10% calcium chloride solution per ml of plasma [34]. The activated F2 fraction was used to moisten the previously with sterile saline rehydrated and subsequently with sterile cotton pellets dried mineralized cancellous bone particulate allografts as well as the pericardium membrane used in this study.

The patient was prepared for surgery and anesthetized via local infiltration (Ultracain-DS Forte, Sanofi-Aventis, Frankfurt/Main, Germany). Using an atraumatic surgical technique, the non-restorable tooth was extracted with extreme care to preserve the alveolar bone surrounding the tooth. Curettage of the extraction site was performed to remove all soft tissue debris and granulation tissue, and to stimulate bleeding from the osseous base to promote healing. The rehydrated allograft was loosely packed into the prepared extraction socket and intentionally overextended up to the cemento-enamel junction (CEJ) or the restorative margin (RM) of the adjacent teeth in order to augment an already occurred bone loss at the adjacent tooth as well as to compensate for a potential graft resorption. Afterwards the site was covered with a resorbable native pericardium membrane (Jason^®^ membrane, botiss biomaterials GmbH, Germany) in order to promote new bone formation by excluding epithelial migration into the graft site. A coronally advanced flap with a periosteal incision [38, 39] was surgically performed, and primary closure without tension was achieved using vertical and horizontal cross mattress sutures (Gore-Tex Suture, W.L. Gore and Associates, Inc., Flagstaff, AZ, USA) (Fig. 5a–i).

#### Surgical phase 2: dental implant placement (T3)

Four months after tooth extraction and ridge preservation procedures, the patient was reappointed and prepared for dental implant placement surgery. Anesthesia was induced via local infiltration and venous blood was drawn as described above. An osteotomy for implant placement was prepared in the alveolar bone by sequential cutting with surgical drills in graduated diameters using a minimal invasive surgical technique. Implants of two different manufactures were placed according to the instructions provided by their respective manufacturers. The patients were randomly assigned to one of the implant systems used as described above.

The implants were moistened with the PRGF-2 liquid and placed approximately 3 mm below the CEJ or the RM of the adjacent teeth, the fixture mounts were removed from the implants and system-specific components were placed in order to cover the implant and to prevent internal debris contamination.

#### Surgical phase 3: uncovering of the dental implant (T4)

Four months after implant placement and 8 months following ARP, the patient was reappointed and prepared for dental implant uncovery surgery. Anesthesia was induced via local infiltration as described above. A minimally traumatic approach was used to locate the implant and to remove the closure screw. Implants were internally rinsed thoroughly using a 2.0% chlorhexidine rinse and the healing abutments were placed according to the manufacturer’s instructions.

### Postoperative instructions

Following surgical procedures, analgesics (paracetamol 800 mg, t.i.d.) and prophylactic antibiotics (amoxicillin 500 mg, t.i.d., only after surgical phase 1 and 2) were prescribed for 7 days postoperatively [40, 41]. However, the need for postoperative antibiotics is controversially discussed in the literature and a single prophylactic dose of antibiotics should also be considered [42].Tooth brushing in the surgical area was limited for the first 2 weeks. In addition, 0.2% chlorhexidine mouthwash (Chlorhexamed forte, Glaxo Smith Kline, Brentford, UK) was prescribed 3 times daily for 1 min in order to maintain the oral flora and prevent infection. Sutures were removed 10 days postoperatively and routine monitoring appointments were held at monthly intervals to evaluate healing.

### Implant restoration and supportive care (T5–T8)

Approximately 4–8 weeks following uncovery of the implants, the final implant restorations were inserted at the individual referring dental office. Eleven implant restorations (30.6%) were screw-retained and 25 implant restorations (69.4%) were cement-retained. All screw-retained restorations were located at posterior sites. The patients were scheduled for supportive periodontal maintenance care at their referring dental office and our periodontal office in an alternating manner in 3–4 months intervals throughout the study period. At the first visit following implant restoration (T5), clinical and radiographic data were obtained as baseline measurement. During the observational period those data were again recorded at 1 year (T6), 2 years (T7), and 3 years (T8) of follow-up and statistically evaluated in order to detect statistically significant changes of ABC-M and ABC-D levels as well as the clinical variables at the different follow-up time points (T5–T8).

### Clinical evaluation

Clinical parameters around the implant restoration were obtained following implant restoration at T5 through T8 using a calibrated implant probe (Hu-Friedy Mfg. Co., LLC. European Headquarters Astropark Lyoner Str. 9. D-60528 Frankfurt am Main Germany, Global Headquarters, Chicago, Illinois, USA) for the recording of 6-point pocket probing depth (PPD) as well as bleeding on probing (BOP), and peri-implant recession defects (REC). Clinical measurements were obtained by one experienced blinded clinician.

### Radiographic evaluation

**Fig. 2:**
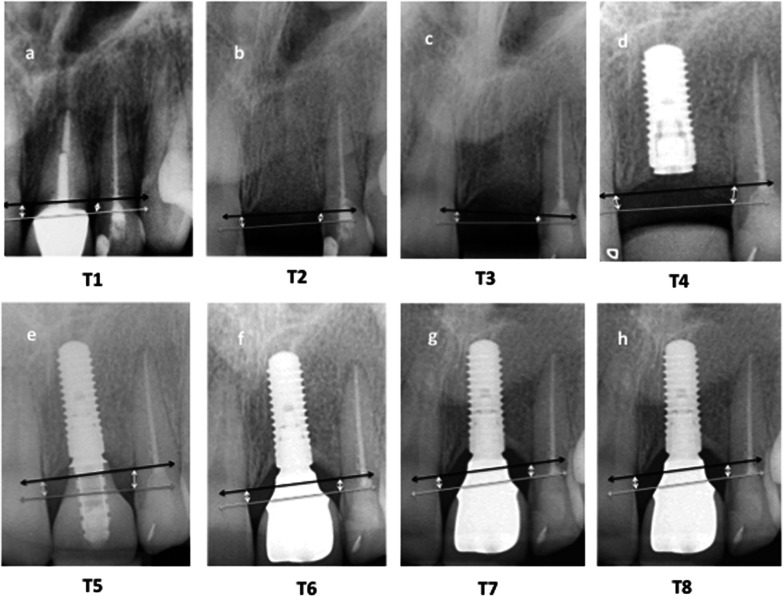
**a–h** This figure demonstrates the measurement technique at the mesial (ABC-M) and the distal (ABC-D) aspects of the alveolar crest throughout the observational period between T1 (**a** following tooth extraction)–T8 (**h** 3 years following restoration) for a bone-level implant

**Fig. 3:**
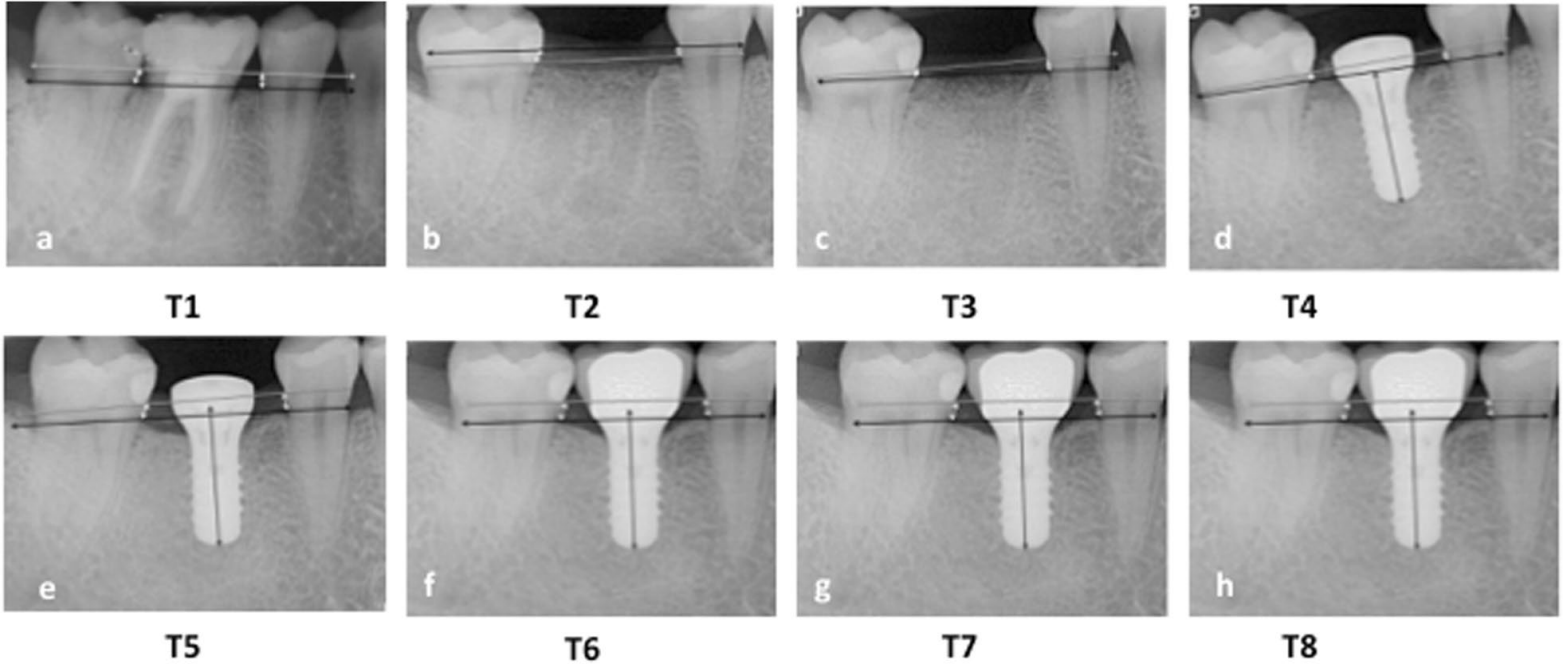
**a–h** This figure demonstrates the measurement technique at the mesial (ABC-M) and the distal (ABC-D) aspects of the alveolar crest throughout the observational period between T1 (**a** following tooth extraction)–T8 (**h** 3 years following restoration) for a tissue-level implant

Periapical digital radiographs were taken at the eight different time points as described below by the same radiographically trained person in the same specialty dental office using a Rinn XCP X-ray holder and a Carestream CS 2200 X-ray unit (Carestream, Rochester New York, USA). Measurements were performed with the Carestream Dental Imaging Software 6.13.1. From T1 to T3 the calibration of the software was performed using the known mesio-distal width of the adjacent teeth, from T4 onwards, the calibration was performed according to the known implant length. Radiographs were taken at eight time points: T1–T8 (Fig. 1). To evaluate alveolar bone change, the distance from the alveolar bone crest (ABC) to the cemento-enamel junction (CEJ) or restorative margin (RM) of the adjacent teeth was measured on all periapical radiographs using digital software [43]. Specifically, measurements were made along a vertical line that extended parallel to the long axis of the tooth or implant from the ABC to the CEJ or RM on the distal surface of the mesial adjacent tooth (ABC-D), the mesial surface of the distal adjacent tooth (ABC-M), and on the mesial and distal surfaces (ABC-M + ABC-D) of both the tooth to be extracted and on the replacement dental implant restoration (Fig. 2a–h for tissue-level implants and Fig. 3a–h for bone-level implants). Changes in ABC to CEJ or ABC to RM distances were thus indicative of bone loss or bone gain [43]. The radiographic evaluation was obtained by one experienced blinded clinician.

### Statistical analysis

For the power analyses, sample size was predicated on assuring that the primary study objective had adequate power to perform our analyses. A sample size of 11 patients per group (number of groups = 2) (total sample size = 22 patients) was the required sample to be statistically significant with 80% power and at a significance level of 95% (accepted α error = 0.05). Sample size per group did not need to be increased to control for attrition bias. The sample size was calculated using G power software and derived from our previous study [44]. Therefore, a sample size of 18 patients in each treatment group was considered to be sufficient to meet the primary objective of this study. For continuous data, the mean, standard deviation (SD), as well as the minimum and maximum were calculated.

The data were anonymized and statistical analysis was obtained by a statistician blinded to the individual bone-grafting material and implant brand used. Statistical software (IBM SPSS Statistics for Windows, Version 22.0, Turkey; IBM Corp., Armonk, NY, USA) was used for all statistical analyses of the data obtained in the study. Conformity of the parameters to normal distribution was assessed by the Shapiro–Wilk test. Descriptive statistical methods (mean, median, standard deviation) and comparisons of quantitative data were performed using the one-way Anova test for intergroup comparisons of parameters with normal distribution, and Turkey HDS test for the determination of the group causing difference. Kruskal–Wallis test was used for intergroup comparisons of parameters without normal distribution, and Mann–Whitney *U* test was used for the determination of the group causing difference. Paired sampled *t*-test was used for in-group comparisons of parameters with normal distribution. Chi-square test was used for comparison of qualitative data. Significance was evaluated at a level of *p* < 0.05.

## Results

36 patients (17 women and 19 men) with a mean age of 53 years (range 38–66 years) for group 1 (maxgraft^®^) and 55 years (range 33–75 years), for group 2 (Puros^®^), respectively, were enrolled in the present study. All of them completed the treatment and no patient was lost during follow-up. All patients were non-smokers and free of any systemic disease. In total, 36 teeth were extracted: 13 central and lateral incisors (36.1%); 9 premolars (25%); and 14 molars (38.9%). In group 1 the allocation of the teeth was as follows: six incisors (46.1%), five premolars (55.5%), and seven molars (50%). In group 2, seven incisors (53.9%), five premolars (44.5%), and seven molars (50%) were extracted. Pre-extraction clinical evaluation revealed mobility in 21 teeth (58.63%), furcation defects in 9 teeth (25%), occlusal contact in 35 teeth (97.2%) and endodontic treatment in 26 teeth (72.2%). A total of 36 dental implants from 2 manufacturers were placed 4 months after extraction and grafting: 13 Astra Tech Implants (group 1: six, group 2: seven) and 23 Straumann implants [11 tissue-level, (group 1: six, group 2: five), and 12 bone-level Implants, (group 1: six, group 2: six)]. The distribution of implants placed included 13 in central and lateral incisor regions, 9 in premolar areas, and 14 in molar locations. Neither implant or graft failures nor adverse events occurred. Only two patients showed a prolonged healing phase, which, however, did not cause any complications affecting the final outcome.

### Changes of alveolar bone crest levels during surgical treatment phase (T1–T4)

**Fig. 4:**
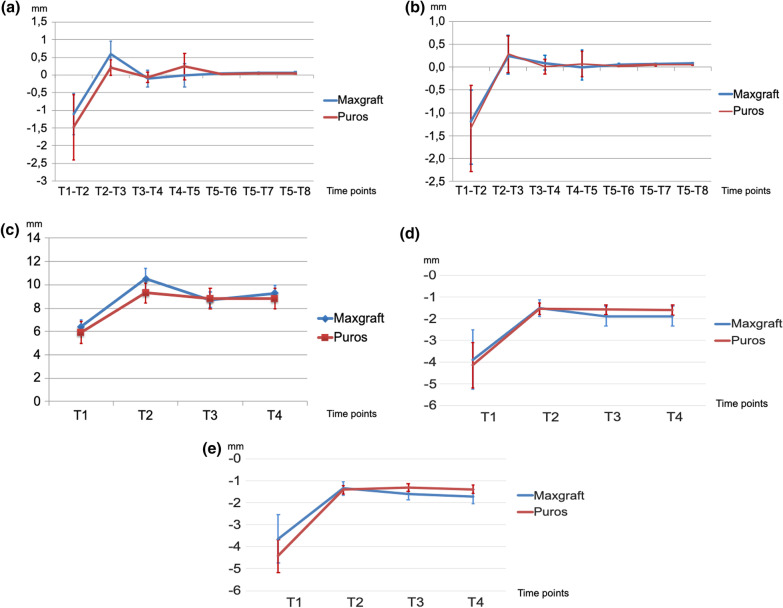
**a** This figure shows changes in the measurements of the distance of alveolar bone crest (ABC) to the cemento-enemal junjction (CEJ) or the restorative margin (RM) for the **mesial** aspect by evaluation time [T1 (after tooth extraction), T2 (after ARP), T3 (4 months later at implant placement), T4 (4 months later at implant uncovery) and T5 (after implant restoration)] as well as at 1 (T5–T6), 2 (T5–T7), and 3 years (T5–T8) of clinical functioning compared by bone-grafting material. **b** This figure shows changes in the measurements of the distance of alveolar bone crest (ABC) to the cemento-enemal junjction (CEJ) or the restorative margin (RM) for the **distal** aspect by evaluation time [T1 (after tooth extraction), T2 (after ARP), T3 (4 months later at implant placement), T4 (4 months later at implant uncovery) and T5 (after implant restoration)] as well as at 1 (T5–T6), 2 (T5–T7), and 3 years (T5–T8) of clinical functioning compared by bone-grafting material. **c** This figure shows changes in the clinical measurement values for the **bucco-lingual bone width** (ABC-BL) by evaluation time [(T1 (after tooth extraction), T2 (after ARP), T3 (4 months later at implant placement), T4 (4 months later at implant uncovery)] by bone-grafting material. **d** This figure shows changes in the clinical measurement values for the **buccal bone height **(ABC-B) by evaluation time [(T1 (after tooth extraction), T2 (after ARP), T3 (4 months later at implant placement), T4 (4 months later at implant uncovery)] by bone-grafting material. **e** This figure shows changes in the clinical measurement values for the **oral bone height** (ABC-O) by evaluation time [(T1 (after tooth extraction), T2 (after ARP), T3 (4 months later at implant placement), T4 (4 months later at implant uncovery)] by bone-grafting material

The changes of alveolar bone crest levels throughout T1–T4 are due to bone augmentation at the time of tooth extraction as well bone resorption processes during healing. Initially, following careful extraction of the tooth, bony defects of the socket walls at the mesial and distal aspects, the bucco-lingual width as well as the buccal and oral alveolar bone crest height were observed and recorded (see below for a detailed description). This resulted in a distribution of six 2-wall defects and twelve 3-wall defects for group 1 and five 2-wall defects and thirteen 3-wall defects for group 2, respectively. The mean changes of alveolar bone crest levels at the mesial and distal aspects between the different timepoints T1–T5 before implant restoration and 1 year (T5–T6), 2 years (T5–T7), and 3 years (T5–T8) after implant restoration are shown in Table 2 and Fig. 4a and b, the mean changes of alveolar bone crest levels at the bucco-lingual aspect between the different timepoints T1–T4 before implant restoration are shown in Table 2 and Fig. 4c, the corresponding standard deviations are shown in Table 3. The mean changes of alveolar bone crest levels at the buccal and oral aspect between the different timepoints T1–T4 before implant restoration are shown in Table 2 and Fig. 4d, e, the corresponding standard deviations are shown in Table 3.

### Changes of alveolar bone crest levels during surgical treatment phase (T1–T2)

Changes in mesial (ABC-M) and distal (ABC-D) as well as bucco-lingual (ABC-BL) and buccal (ABC-B) and oral (ABC-L) alveolar bone crest levels showed statistically highly significant differences between T1 (preoperatively) and T2 (immediately postoperatively) for both bone allografts (maxgraft^®^: *p* = 0,0001 mesial, *p* = 0,002 distal, *p* = 0.001 bucco-lingual, *p* = 0.0014 buccal, *p* = 0.0001 oral and Puros®: *p* = 0.001 mesial, *p* = 0.008 distal, *p* = 0.001 bucco-lingual, *p* = 0.0002 buccal, *p* = 0.0001 oral, respectively), which is due to the bone augmentation of the site after tooth extraction (Table 1, Fig. 4a–e). The amount of bone augmentation varied between bone allografts and mesial, distal, buccal, and oral aspects. Specifically, the mean amount of bone augmentation in the maxgraft^®^ group was for ABC-M: 2,77 mm (min: 0.95 mm—max.: 5.61 mm, STD: 1.57 mm), and for ABC-D: 2,51 mm (min: 0.16 mm—max.: 6.79 mm, STD: 1.49 mm), for ABC-BL: 6.42 mm (min.: 0,57 mm—max.: 7.70 mm, STD: 0.57 mm), for ABC-B: 3.83 mm (min.: 1.15 mm—max. 10.55 mm, STD: 2.80 mm), and for ABC-O: 3.55 mm (1.16 mm—max.: 7.56 mm, STD: 2.07 mm), respectively. The mean amount of bone augmentation in the Puros^®^ group was for ABC-M: 3,49 mm (min: 0.93 mm—max.: 11.25 mm, STD: 2.32 mm), and for ABC-D: 3.11 mm (min: 1.61—max.: 8.46 mm, STD: 1.71 mm), and for ABC-BL: 5.91 mm (min.: 0,95 mm—max.: 7.30 mm, STD: 0.95 mm), for ABC-B: 4.03 mm (min.: 1.24 mm—max. 9.57 mm, STD: 2.15 mm), and for ABC-O: 4.26 mm (1.54 mm—max.: 7.25 mm, STD: 1.54 mm), respectively (Table 2, Fig. 4a–e).

**Table 1:** Shows the significant and not-significant changes (p-value) in the mesial, distal, bucco-lingual, buccal, and oral dimensions of the alveolar ridge (ABC-M, ABC-D, ABC-BL, ABC-B, and ABC-O) in relation to the different time points (T1–T8)

ABC-M p-values*							
Timepoints	T 1/2	T 2/3	T 3/4	T 4/5	T 5/6	T 5/7	T 5/8
Maxgraft	**0.0001**	**0.002**	0.409	0.930	0.645	0.254	0.219
Puros	**0.001**	0.076	0.204	0.240	0.623	0.392	0.183
ABC-D *p*-values*							
Maxgraft	**0.002**	0.246	0.253	0.167	0.567	0.317	0.267
Puros	**0.008**	0.153	0.942	0.575	0.692	0.293	0.196
ABC-BL *p*-values*							
Maxgraft	**0.001**	0.001	0.231				
Puros	**0.001**	0.233	1				
ABC-B *p*-values*							
Maxgraft	**0.0014**	0.1952	0.9778				
Puros	**0.0001**	0.7462	0.9511				
ABC-O *p*-values*							
Maxgraft	**0.0001**	0.2271	0.5632				
Puros	**0.0002**	0.4806	0.5752				

*ANOVA Tukey’s HSD test for comparison of Maxgraft^®^ vs. Puros^®^

**Table 2:** Shows the radiographically detectable mean changes (mm) from the mesial (ABC-M) and distal (ABC-D) alveolar bone crest to the cemento-enamel junction (CEJ) or restorative margin (RM) between the different time points at T1–T2, T2–T3, T3–T4, T5–T6, at 1 (T5–T6), 2 (T5–T7), and 3 years (T5–T8) of clinical functioning as well as the clinically detectable mean changes of alveolar crest width (ABC-BL) and for alveolar bone crest height (ABC-B and ABC-O) for T1–T4 compared by bone-grafting material

ABC-M mean change in mm							
Timepoints	T 1–2	T 2–3	T 3–4	T 4–5	T 5–6	T 5–7	T 5–8
Maxgraft	+ 1.11	− 0.6	+ 0.1	+ 0.01	− 0.04	− 0.06	− 0.07
Puros	+ 1.48	− 0.21	+ 0.07	− 0.24	− 0.03	− 0.04	− 0.05
ABC-D mean change in mm							
Maxgraft	+ 1.18	− 0.25	− 0.09	− 0.25	− 0.06	− 0.07	− 0.08
Puros	+ 1.34	− 0.28	− 0.01	− 0.07	− 0.03	− 0.05	− 0.06
ABC-BL mean change in mm							
Maxgraft	+ 4.1	− 1.81	+ 0.56				
Puros	+ 3.4	− 0.49	± 0				
ABC-B mean change in mm							
Maxgraft	+ 2.36	− 0.36	− 0.01				
Puros	+ 2.6	− 0.05	+ 0.01				
ABC-O mean change in mm							
Maxgraft	+ 2.24	− 0.23	− 0.11				
Puros	+ 3.91	+ 0.13	− 0.08				

### Changes of alveolar bone crest levels during surgical treatment phase (T2–T3)

The differences in alveolar bone crest changes between T2 (immediately postoperatively) and T3 (4 months postoperatively, time of implant placement) showed the amount of resorption of the allograft materials during 4 months of healing between bone augmentation (T2) and implant placement (T3). The changes at the ABC-M levels of the maxgraft^®^ group showed a statistically significant difference (*p* = 0.002, min: 0.00 mm—max.: 3.0 mm, STD: 0.81 mm at T2 versus min: 0.48 mm—max.: 3.83 mm, STD: 0.89 mm at T3, respectively), and the changes at the ABC-BL levels of the maxgraft^®^ group showed a statistically significant difference (*p* = 0.001, min: 0.91 mm—max.: 12.3 mm, STD: 0.91 mm at T2 versus min: 0.67 mm—max.: 10,10 mm, STD: 0.67 mm at T3, respectively) all other changes in ABC-M, ABC-D, ABC-B, ABC-O, and ABC-BL levels for both materials showed no statistically significant differences throughout the observational period (Tables 1, 2, 3, Fig. 4a–e).

**Table 3:** Shows the standard deviation (mm) of changes from the mesial (ABC-M) and distal (ABC-D) alveolar bone crest to the cemento-enamel junction (CEJ) or restorative margin (RM) between the different time points at T1–T2, T2–T3, T3–T4, T5–T6, at 1 (T5–T6), 2 (T5–T7), and 3 years (T5–T8) of clinical functioning as well as for the clinically detectable mean changes of alveolar crest width (ABC-BL) and for alveolar bone crest height (ABC-B and ABC-O) for T1–T4 compared by bone-grafting material

ABC-M STD in mm							
Timepoints	T 1–2	T 2–3	T 3–4	T 4–5	T 5–6	T 5–7	T 5–8
Maxgraft	1.18	0.69	0.48	0.66	0.01	0.03	0.03
Puros	1.83	0.44	0.29	0.73	0.02	0.03	0.03
ABC-D STD in mm							
Maxgraft	1.35	0.89	0.33	0.73	0.03	0.03	0.04
Puros	1.88	0.79	0.32	0.55	0.04	0.04	0.03
ABC-BL STD in mm							
Maxgraft	0.91	0.67	0.66				
Puros	0.86	0.88	0.88				
ABC-B STD in mm							
Maxgraft	2.03	0.17	0.1				
Puros	1.59	0.09	0.03				
ABC-O STD in mm							
Maxgraft	1.81	0.31	0.08				
Puros	1.18	0.04	0.01				

*STD* standard deviation

### Changes of alveolar bone crest levels during surgical treatment phase (T3–T4)

The differences in alveolar bone crest changes between T3 (implant placement) and T4 (implant uncovery) showed the potential amount of resorption of the allograft materials during 4 months of healing between implant placement (T3) and implant uncovery (T4). No statistically significant changes were obtained in ABC-M, ABC-D, ABC-B, ABC-O, and ABC-BL levels for both materials (Tables 1, 2, 3, Fig. 4a–e).

The differences in alveolar bone crest changes between T4 (implant uncovery) and T5 (implant restoration) varied in time between 3 and 8 weeks due to the amount of time needed in the individual dental office and the corresponding dental laboratory for the production of the final restoration. No statistically significant changes in ABC-M or ABC-D levels for both materials were recorded (Tables 1, 2, 3, Fig. 4a, b), measurements for ABC-B, ABC-O, and ABC-BL were not obtained for T5–T8 since nor further surgical re-entries were carried out.

### Changes of alveolar bone crest levels during follow-up period (T5–T8)

The differences in alveolar bone crest changes between T5 (implant restoration) and T6 (12 months following implant restoration) showed the potential amount of bone resorption at ABC-M and ABC-D during 12 months of functioning of the individual implant. No statistically significant changes in ABC-M or ABC-D levels for both materials were obtained (Tables 1, 2, 3, Fig. 4a, b).

The differences in alveolar bone crest changes between T6 (12 months following implant restoration) and T7 (24 months following implant restoration) showed the potential amount of bone resorption at ABC-M and ABC-D during the 1st year and the 2nd year of clinical functioning of the individual implant. No statistically significant changes in ABC-M or ABC-D levels for both materials were found (Tables 1, 2, 3, Fig. 4a, b).

The differences in alveolar bone crest changes between T7 (24 months following implant restoration) and T8 (36 months following implant restoration) showed the potential amount of bone resorption at ABC-M and ABC-D during the 2nd and the 3rd year of clinical functioning of the individual implant. No statistically significant changes in ABC-M or ABC-D levels for both materials were observed (Tables 1, 2, 3, Fig. 4a, b).

Furthermore, the analysis for T5 versus T6 (1 year of clinical functioning), T5 versus T7 (2 years of clinical functioning) as well as the statistical analysis for T5 versus T8 (3 years of clinical functioning) did not show any statistically significant differences in ABC-M or ABC-D levels during the observed periods, neither within the individual groups of bone allograft materials, nor in between the two groups (Tables 1, 2, 3 and Fig. 4a, b).

### Summary of results of alveolar bone-level changes at T1–T8

In summary, the findings in both groups showed a statistically highly significant difference between ABC-M, ABC-D, ABC-B, ABC-O, and ABC-BL levels at T1–T2 (bone augmentation) as well as a significant change in ABC-M and ABC-BL levels at T2–T3 (implant placement after 4 months of healing) for the maxgraft^®^ group (*p* = 0.001, Fig. 4a, c–e). There were no statistically significant differences between ABC-M and ABC-D levels at T3 through T8 (*p* ˃ 0.05) (Tables 1, 2, 3, Figs. 4a, b) and at the comparison of ABC-M and ABC-D level changes at 1 year (T5–T6), 2 years (T5–T7), and 3 years (T5–T8) of clinical functioning of the implant restoration within and in between the bone allograft groups (Tables 1, 2, 3, Fig. 4a, b). No failures of the bone augmentation/ARP procedures and no adverse events were observed throughout the observational period.

**Fig. 5:**
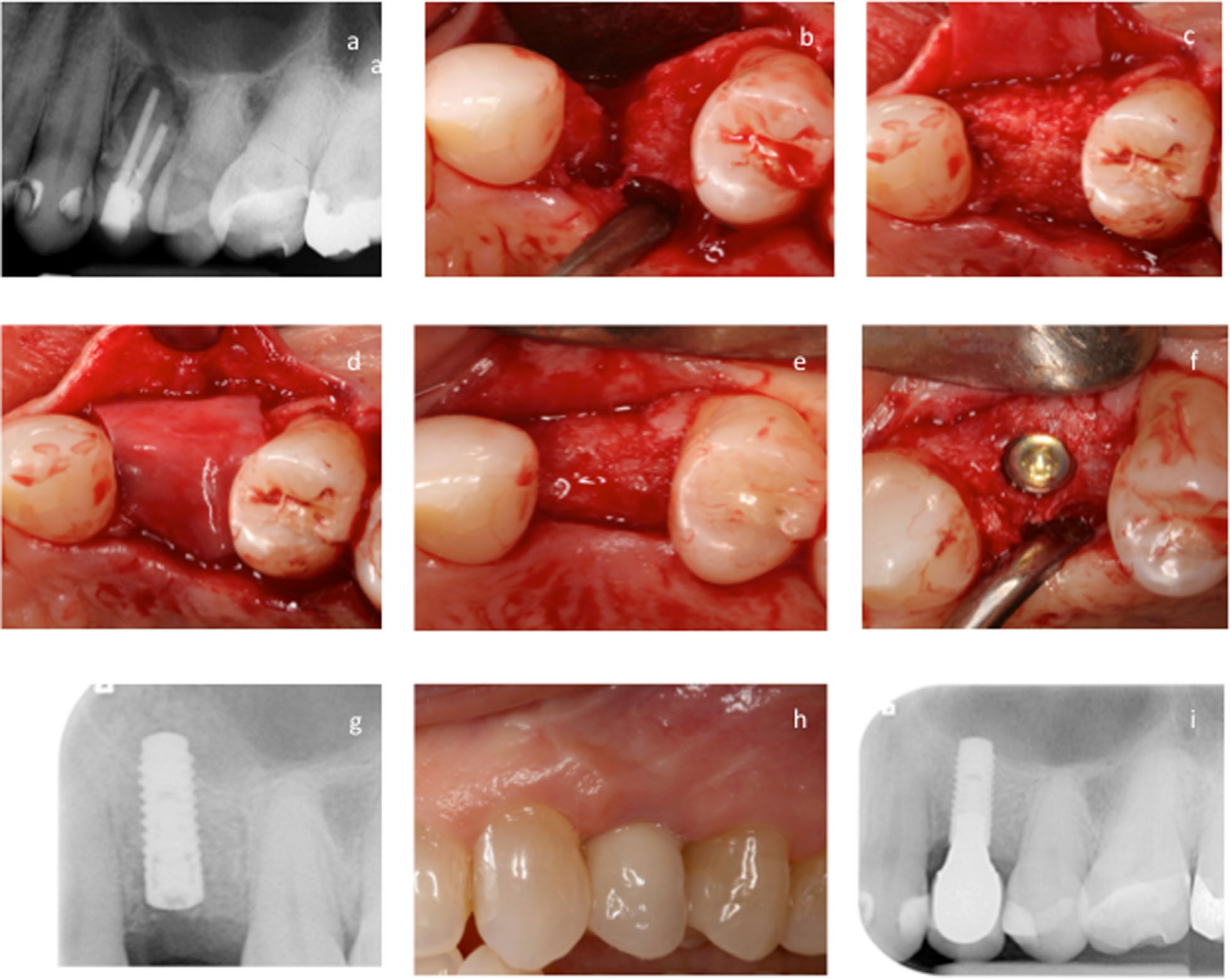
**a**–**i** Shows the different steps of the socket preservation procedure. The initial radiograph (**a**) demonstrates a non-restorable tooth #12. The clinical appearance of the bony defect following careful extraction of the tooth is shown in **b**. **c** shows the augmented alveolar crest with a bone allograft material mixed with F-2 of the PRGF system and covered with a pericardium membrane (**d**). The clinical view after 4 months of healing is visible in **e**, at time of implant insertion (**f**). The radiograph after implant insertion (**g**), the clinical view of the final restoration (**h**), and the final radiograph 3 years following implant restoration (**i**) are shown

### Additional clinical variables and implant survival rates

There were no failures of the implants observed and no adverse events were recorded. The implant survival rate was 100% throughout the observational period. No statistically significant differences were observed within or in between the allograft groups regarding the implant survival rates, implant brands, implant lengths, implant diameters, the tissue- or bone-level type of the implants placed, the type of retention of the crowns, initial tooth mobility, endodontic status of the extracted tooth, periodontal status, furcation involvement, or occlusal contacts of the replaced teeth (*p* ˃ 0.05, data not shown). Furthermore, no statistically significant differences were found for the clinical parameters like PPD, BOP, and REC throughout the observational period (*p* > 0.05, data not shown).

## Discussion

A universal agreement on the factors constituting peri-implant marginal bone loss, a universal recommendation how it should be measured, or when the initial baseline measurement should be made, does currently not exist. Changes in marginal bone levels around implants remain controversially discussed in the dental literature [45]. For example, baseline measurements in early implant studies were conducted at abutment connection, but only bone loss below the implant’s neck, which was countersunk of approximately 2.0 mm below the marginal bone level, was measured [46]. Bone loss from the crest of the ridge and bone loss before prosthetic loading were initially excluded [46]. Furthermore, researchers continued to use abutment connection for the baseline measurement, but calculated bone loss from a fixed location on the implant (e.g., RM) to the location of the first bone contact with the implant surface [43]. This meant that the peri-implant bone loss that was greater than the location of the first bone contact with the implant surface (e.g., saucerization around the implant) was not included in the bone loss calculations [47]. Since the concept of immediate loading of dental implants has become more widespread, baseline measurements were often made immediately after provisionalization on the day of implant placement at the location of the first bone to implant contact at the implant surface. This is still used as an appropriate measuring point of bone loss by some researchers [48].

However, in the present study alveolar bone-level changes from ABC-M and ABC-D locations to the CEJ or RM of adjacent teeth were measured and evaluated, which is a well-established technique widely reported in the periodontal, pediatric, anthropologic, and forensic dental literature [49–51]. As described before, periapical radiographs were taken at 8 time points in order to evaluate alveolar bone-level changes as the distance from the alveolar bone crest (ABC) to the cemento-enamel junction (CEJ) or restorative margin (RM) of the adjacent teeth using digital software [43]. Furthermore, clinical measurements for the changes of alveolar crest width (ABC-BL) as well as alveolar bone crest height (ABC-B, ABC-O) were recorded between the timepoints T1–T4 (initial augmentation to implant uncovery). This was carried out in order to detect potential changes of the buccal plate of bone which is known to be subject to early resorption processes [32, 52–54]. The recordings for PPD, BOP, and REC around the implants and the adjacent teeth from the time of implant restoration (T5) throughout the observational period (T5–T8) were collected in order to account for potential radiographically underestimated changes in ABC at the buccal and palatal/lingual aspects of the implants, since 2-dimensional periapical radiographs were used. Due to reduction of radiographic exposure and costs for the patients no 3-dimensional CBCT scan evaluation was obtained.

With regard to the surgical procedure of ARP, a systematic review revealed that wound closure, the use of a membrane and the application of a xenograft or an allograft resulted in better outcomes than unassisted healing, showing a mean effect of 1.9 mm in terms of bucco-lingual width and 2.1 mm for the mid-buccal height [55]. These findings were confirmed by a recent systematic review investigating the amount of horizontal ridge resorption with different bone substitute materials compared to unassisted healing, resulting in 1.52 mm (SD 1.29) for allogeneic material and 3.1 mm (SD 1.07) for unassisted healing, respectively [56].

In humans, dimensional alterations have been reported to cause a ridge width reduction of up to 50% during the first year following tooth loss in premolar and molar sites, where two-thirds of the total changes take place within the first 3 months post-extraction [57]. A systematic review showed a loss of 2.6–4.5 mm in width and 0.4–3.9 mm in height of healed sockets [58]. ARP via socket grafting attenuates the physiological bone dimensional changes that typically follow tooth extraction and may therefore prevent 1.5–2.4 mm of horizontal, 1–2.5 mm of vertical mid-buccal and 0.8–1.5 mm of mid-lingual vertical bone resorption as compared to tooth extraction alone [59]. It is very well established that placement of a barrier membrane over the graft material can influence the amount of newly formed vital bone by excluding epithelial cells during the critical early phase of bone healing. Furthermore, Eskan et al. demonstrated that the combination of an allogeneic bone-grafting material with platelet-rich plasma covered with a resorbable membrane showed superior results compared to allogeneic bone-grafting material and a membrane alone [60].

This supports our findings very well and all augmented extraction sockets achieved vertical gain in bone volume, regardless of the allograft group (Tables 1, 2, 3). The only statistically significant differences appeared for both groups for the comparison between T1 and T2 (Tables 1, 2, 3) (*p* < 0.05). This was possibly due to an intentional over-augmentation of the socket in order to augment already lost bone volume, especially at the buccal aspect of the extraction socket, and to compensate for a potential resorption of the graft. The mean change of ABC-M/ABC-D levels were 2.77 mm/2.52 mm for the maxgraft^®^ and 3.4 mm/3.11 mm for the Puros^®^ group, respectively. However, the minimum and maximum change of ABC levels within these groups varied between 0.16 and 11.25 mm, which demonstrates a significant amount of preserved and augmented ABC volume.

The only significant difference at T2–T3 occurred in the ABC-M and ABC-BL level of the maxgraft^®^ group which can either be explained by a greater amount of initial over-augmentation, or a higher rate of initial graft resorption. The increase of ABC-BL for the maxgraft^®^ group between T3-T4 was due to additional bone grafting at time of implant placement in order to compensate for the resorption between T2–T3 (Fig. 4c). In the Puros^®^ group no dimensional changes were observed and therefore, an additional grafting needed to be carried out. However, the comparison of ABC-M and ABC-D levels throughout the observation period of 3 years following implant restoration revealed no further significant changes between the two allograft groups tested, or within each individual group. Furthermore, the mean values of ABC-M and ABC-D levels between the groups and within the groups became almost identical throughout the observational period (Fig. 4a, b).

Our findings are in agreement with other investigations showing comparable clinical results in terms of healing and incorporation of bone allograft materials [61].

To our knowledge, there is very limited information in the literature available regarding the long-term clinical parameters of implants placed into socket preserved areas. In a recent study, Wessels et al. compared the 5-year clinical outcome of early implant placement with simultaneous GBR and alveolar ridge preservation and late implant placement [62]. The results indicate that ARP and late implant placement led to a significantly higher predictability in terms of esthetic outcome using the pink esthetic score [63]. The clinical and esthetic outcomes for single-implant restorations following ARP and connective tissue graft were investigated in a prospective clinical trial [64]. The 5-year results were very favorable, however, there was no control group investigated in this study.

Therefore, the present randomized controlled clinical trial represents one of the very few clinical studies with a follow-up period of several years.

Both allogeneic bone-grafting materials used in the present study were treated in a multi-step chemical cleaning process in order to inactivate potential pathogens. Maxgraft^®^ is finally dehydrated by freeze-drying and is pooled from multiple donors, whereas Puros^®^ is solvent-dehydrated prior to packaging and gamma-irradiation and is harvested from a single donor. Each process has been validated to inactivate viruses and bacteria (comprising delipidization, osmotic and oxidative treatment, dehydration, and sterilization through limited-dose gamma radiation), which prevents disease transmission by removing cells, viruses, antigens, and pathogens [65]. During this process the natural collagen–bone mineral composition is preserved and they provide osteoconductive type 1 collagen, which is a natural substrate for the support and growth of a variety of cells and tissues in the body [66], and osteoinductive bone morphogenic proteins (BMPs) that encourage new bone formation [67].

In a previous histological and immunohistochemical study, we demonstrated that the allogeneic bone-grafting materials used in the present study showed equivalent results of clinical outcome, bone formation and lack of immunological potential in alveolar ridge augmentation procedures [26].

We are aware of the fact that studying only 16 subjects per grafting material does not allow for a generalized statement and that the method of analyzing 2-dimensional periapical radiographs carries the potential for under- and over-estimation of the individual ABC levels, especially at the mid-buccal and mid-palatal/lingual aspects of the implant restorations. However, the performance of 3-dimensional CBCT scans would have significantly increased the dosage of radiographic exposure as well as costs for the patients. Therefore, we tried to compensate for that limitation by the inclusion of intraoperative measurements of the bucco-lingual width and the buccal and oral height of the alveolar crest (T1–T4), as well as clinical parameters like PPD, BOP, and REC at the implant restorations and adjacent teeth in a follow-up period of 3 years (T5–T8). Furthermore, the use of two different implant systems might be regarded as a limitation of this study. However, this is due to fact that in a referral-based specialty practice the system of choice of the referring dentist was used and since there were no statistically significant differences observed between the two implant systems the importance of this variable on the outcome of the surgical procedure could be regarded as very limited. Nevertheless, our work may serve as a clinical pilot study with a promising outcome and confirms the results of already published data using the same allograft materials for the augmentation of maxillary sinuses and alveolar ridges.

## Conclusions

Within the limitations of the present study the data clearly suggest that immediate augmentation of fresh extraction sockets with mineralized cancellous bone allograft and a pericardium membrane could be a successful treatment option for the preservation and the augmentation of post-extraction ridges for implant placement. In our study, implants and adjacent teeth demonstrated increased alveolar bone crest levels through 1–3 years of clinical function. Clinicians may consider ARP in clinical scenarios in which minimizing alveolar ridge dimensional changes or simultaneous augmentation of the damaged alveolar ridge is critical. Such scenarios could include extraction sites in areas of esthetic priority when an implant-supported restoration is planned as well as in extraction sites on which major ridge reduction is expected and may jeopardize implant placement, due to a thin and/or substantially damaged buccal plate of bone. Furthermore, on posterior sites exhibiting limited ridge height post-extraction, which may lead to implant proximity to the maxillary sinus or nerve structures.

Again, in order to support our statistical data, analyses of larger sample sizes are required and planned. Nevertheless, our data indicate a statistically significant difference only in the ABC-M and ABC-BL levels after 4 months of healing for the maxgraft^®^ group (T2 vs. T3, *p* < 0.05), otherwise no significant differences between the two tested materials for all parameters were detected and a similar biological behavior after implantation was observed. Therefore, the tested hypothesis could be rejected.

## Data Availability

All data generated or analyzed during this study are included in this published article and its additional information files.

## Abbreviations

ARP: Alveolar ridge preservation
ABC: Alveolar bone crest
ABC-M: Alveolar bone crest-mesial
ABC-D: Alveolar bone crest-distal
ABC-BL: Alveolar bone crest-bucco-lingual
ABC-B: Alveolar bone crest-buccal
ABC-O: Alveolar bone crest-oral
RCT: Randomized controlled trial
RR: Resorption rate
CEJ: Cemento-enemal junction
RM: Restorative margin
PPD: Pocket probing depth
BOP: Bleeding on probing
REC: Recession defect
BMP: Bone morphogenic protein
DFDBA: Demineralized freeze-dried bone allograft
FDBA: Freeze-dried bone allograft
PRGF: Plasma rich in growth factors
